# Attitudes Toward COVID-19 and Seasonal Influenza Vaccines in the Post-COVID Era: A Cross-Sectional Study Among Adults in Malta

**DOI:** 10.3390/pharmacy13040102

**Published:** 2025-07-29

**Authors:** Maria Cordina, Mary Anne Lauri, Josef Lauri

**Affiliations:** 1 Department of Clinical Pharmacology and Therapeutics, WHO Collaborating Centre for Health Professionals Education and Research, Faculty of Medicine and Surgery, University of Malta, MSD 2080 Msida, Malta; 2 Department of Psychology, Faculty of Social Well Being, University of Malta, MSD 2080 Msida, Malta; mary-anne.lauri@um.edu.mt; 3 Department of Mathematics, Faculty of Science, University of Malta, MSD 2080 Msida, Malta; josef.lauri@um.edu.mt

**Keywords:** COVID-19 vaccine, seasonal influenza vaccine, vaccine hesitancy, combined seasonal influenza/COVID-19 vaccine

## Abstract

The uptake of the COVID-19 and seasonal influenza (SI) vaccines have decreased in Europe and especially in Malta. The present study aimed to investigate the attitudes toward COVID-19 and SI vaccines and determine if individuals perceive that these vaccines are relevant to protect their health and identify reasons for their responses. A cross-sectional study using an anonymous questionnaire, informed by the Theory of Planned Behavior, addressing behavior beliefs and attitudes, and targeted at adult residents in Malta, was designed on Google Forms and disseminated using social media between January and March 2024. A total of 555 responses were received. The majority of respondents did not take/intend to take the COVID-19 (75%, *n* = 417) or SI (64.3%, *n* = 362) vaccines, with females being less likely to do so (*p* = 0.033). Perceived lack of safety (31.3%, *n* = 174) was the primary reason for rejecting the COVID-19 vaccine, and perceived lack of a threat from SI (26%, *n* = 144) was the reason for rejecting the SI vaccine. Those having chronic conditions were positively associated with uptake of both vaccines. In the post-pandemic era, these vaccines are not envisaged as having a major role in protecting one’s health. A high degree of skepticism especially toward the combined COVID-19 and SI vaccine in terms of safety, mostly in women, is still present.

## 1. Introduction

The COVID-19 and seasonal influenza (SI) vaccines are both important and necessary tools to protect the health of populations; however, with the decreasing threat from the COVID-19 pandemic, the uptake of these vaccines has diminished [1,2]. Both COVID-19 and seasonal influenza are highly contagious respiratory viruses and have a significant burden on population health and health care systems [3]. COVID-19 vaccines have saved an average of 1.6 million lives in the WHO European Region between December 2020, and March 2023 [4]. WHO recommends that for at-risk groups there should be 75% seasonal influenza coverage since it is responsible for up to 50 million symptomatic cases in the European Union/European Economic Area and 15,000–70,000 deaths due to causes associated with influenza [2]. This is expected to have both public health and economic benefits. Such coverage is projected not only to save lives but also to avoid long term complications as well as decreasing the financial burden on health care systems [5,6]. In 2024, the WHO Regional Office for Europe together with the European Commission and the European Centre for Disease Control and Prevention (ECDC) issued a statement urging vaccination. This statement was made in view of the increased morbidity and mortality, especially in vulnerable groups [2].

Various factors that may have led to the waning in vaccine uptake are possibly at play. One such factor is the COVID-19 pandemic ‘collective amnesia’, where populations want to put the pandemic behind them. The WHO Regional Director for Europe issued a statement warning of the dangers of individuals insufficiently protecting themselves, leading to increased mortality and morbidity especially in vulnerable groups [7]. A second factor is that misinformation and disinformation during the pandemic may also still affect vaccine uptake [8,9]. Even during the COVID-19 pandemic, hesitancy toward both COVID-19 and seasonal influenza vaccines was well documented. A global review covering 114 countries revealed considerable variation in COVID-19 vaccine acceptance across Europe, ranging from as low as 35% in Cyprus and Portugal to as high as 91% in Norway, highlighting significant regional variability and persistent uncertainty in public acceptance [10]. While many studies have looked at determinants of vaccine hesitancy during the pandemic, few have investigated the decrease in vaccine uptake once COVID-19 lost its pandemic status [11,12,13,14].

Malta initially had the fastest COVID-19 vaccine roll out in Europe [15]. Official data demonstrate that following the primary course of COVID-19 vaccines, coverage was over 90% in the adult population [1]. Data gathered by the SHARE study between June and August 2021 placed COVID-19 vaccine coverage for individuals over 50 years of age in Malta as the highest at 98.2% of the population together with Denmark and the highest for SI vaccine coverage at 66.7% [16]. There has then been a steady decrease in COVID-19 vaccine uptake in Malta with only 1.4% of the population receiving the third booster [1]. WHO data show that SIV coverage in Malta in 2023 in older persons was 54.7% [17]. While this is also the case for several other countries in Europe, it is concerning as it falls short of target immunization coverage recommendations issued by the ECDC [1,17,18]. Malta has gone from having one of the highest vaccine coverages for both COVID-19 and seasonal influenza to having one of the lowest.

The present study aimed to follow up on similar previous studies conducted in Malta, while being informed by the Theory of Planned Behavior (TPB) [19,20,21]. The present study, therefore, sought to investigate the attitudes toward COVID-19 and SI vaccines, including the attitudes of significant others that relate to the subjective norm factor in the TPB. This study also attempted to determine if the population still felt that these vaccines were relevant to protect their health and to identify the underlying reasons for their responses.

## 2. Materials and Methods

The study employed a cross-sectional survey design to gather data regarding attitudes and beliefs about the COVID-19 and the SI vaccines in the post-COVID era, targeted at Maltese residents aged 16 years and over. The data were collected between January and March 2024. The anonymous questionnaire was designed on Google Forms and disseminated through popular social media platforms such as Instagram, WhatsApp, Messenger, and Facebook. Respondents were asked to share the questionnaire on their own socials to enhance the response rate.

Eligible participants were informed that the survey took a few minutes to complete and was completely anonymous, participation was voluntary, and that they could stop at any point. Online informed consent was obtained before participants engaged in the survey. Since all respondents were above the age of 16 years, parental consent was not required. The questionnaire was available in both the English and Maltese language, with questions being presented in both languages simultaneously. The questionnaire was piloted with 20 individuals from varied demographic backgrounds, using different electronic devices. Their feedback was used to improve clarity and accessibility. The main issues that needed addressing was ensuring the same visualization and accessibility on the different electronic devices. This was successfully addressed prior to launching the study.

The questions were based on a review of the literature and from knowledge obtained through previous relevant studies carried out in Malta [19,20]. Questions regarding attitudes about the vaccines were loosely based on constructs from the Theory of Planned Behavior [21].

Demographic questions on gender, age at last birthday, educational level, and whether respondents were health care workers (non-specific) or had any chronic/long term conditions were included in the first section.

While it was made clear that the questionnaire was targeted at people residing in Malta, in practice it was possible for anybody with access to answer it. To ensure that the data gathered only originated from Maltese residents, the following question was inserted: What is your country of residence? Response: (i) Malta (ii) other. The selection of ‘others’ prevented any further responses.

As explained above, some questions were loosely based on the Theory of Planned Behavior, while others were stand-alone questions.

Questions for both the COVID-19 and SI vaccines addressed information seeking behavior, where participants were asked to select their main sources of information (TPB-social norm), with the option of selecting multiple sources, beliefs and attitudes, scored on a Likert scale from 1-strongly disagree to 10-strongly agree; degree of agreement with protection provided by the vaccines, encouragement to take the vaccines, the influence of family and friends on vaccination, (TPB-social norm) the value they place on health care professionals’ advice (TPB-social norm), and the ease of obtaining the vaccine (TPB-perceived behavioral control/self-efficacy). Regarding vaccine uptake, they were asked whether they took or intended to take their vaccines for the current season. Those who replied “No” were asked to select one or more answers from a list of pre-determined reasons. Respondents were also asked to rate their degree of willingness to engage in non-pharmaceutical activities (NPAs), such as mask wearing, sanitizing, and social distancing, should another pandemic arise. One additional question addressed the degree of willingness of respondents to take a combined COVID-19 and SI vaccine were it to be made available and to provide reasons for their answer. This question generated a significant wealth of qualitative data.

Data from the questionnaires were downloaded, coded, cleaned, and analyzed using IBM Statistical Package for Social Sciences (SPSS) V26. Questionnaires with incomplete data were discarded. Data gathered were stored in a password-protected database, which could only be accessed by the authors.

Preliminary power analysis to calculate an adequate sample size from a population of 355,156 (population of residents over the age of 16) giving a 95% confidence interval with a 5% margin of error and power of 80% gave a sample size of 384.

Bivariate analysis was used for categorical data together with chi-square tests to determine relationships between demographic data as relevant. Mean/median scores and quartiles were generated for questions related to attitudes and beliefs with regards to COVID-19 and SI vaccines, which were scored on a Likert scale. One-way ANOVA tests and *t*-tests, together with post hoc tests with *p*-values corrected for multiple comparisons using Bonferroni correction, were used to establish relationships between demographics and attitudes and beliefs regarding COVID-19 and SI vaccines as measured on a Likert scale. Assumptions for parametric testing using Shapiro–Wilk test for normality were assessed prior to applying ANOVA tests and *t*-tests. Pearson’s correlation coefficient, r, was used to assess attitudes and beliefs toward the vaccines. The level of significance was set at *p* < 0.05.

The qualitative data generated by the latter part of the question ‘If a combined seasonal influenza and COVID-19 vaccine is available as one vaccine—in one shot—would you be willing to take it? Please give reasons for your answer’ were analyzed using thematic analysis. Following familiarization with the data, similar answers were coded, which were then brought together under subthemes. Related subthemes were grouped into themes. These verbatim quotes were analyzed using thematic analysis with a six-step approach with the aim of identifying themes and subthemes that arise from the raw data. The texts were analyzed by two of the researchers independently and then compared to ensure reliability and validity.

The themes, subthemes, and codes were revisited to ensure that there was no overlap and that codes and subthemes fit well within the themes, thus ensuring that the final themes represented the data.

Ethical clearance was received from the University of Malta Research Ethics Committee UREC (Ethics ID SWB-2024-00013).

## 3. Results

### 3.1. Participants’ Demographic Profile and Vaccine Uptake

Table 1 provides a demographic profile of the 555 participants who were predominantly female (79.9%, *n* = 427), in a relationship (76%, *n* = 422), and having a tertiary or further education (66.1%, *n* = 367). Approximately 75% (*n* = 412) had not taken nor did they intend to take the COVID-19 vaccine. In the case of the SI vaccine, 64.3% (*n* = 362), did not take nor did they intend to take the vaccine. Respondents who rejected to take one of the vaccines were likely to reject the other vaccine (chi-square = 114.6, df = 1, *p* < 0.001). Respondents who were female were less likely to have taken or intended to take the COVID-19 vaccine (chi-square = 8.7, df = 3, *p* = 0.033) or the SI vaccine (chi-square = 6.9, df = 2, *p* = 0.038). Individuals with chronic conditions were more likely to have taken or intend to take both the COVID-19 (chi-square = 11.2, df = 2, *p* = 0.004) and the SI vaccine (chi-square = 10.4, df = 2, *p* = 0.005).

### 3.2. Reasons for Vaccine Rejection

**Figure 1:**
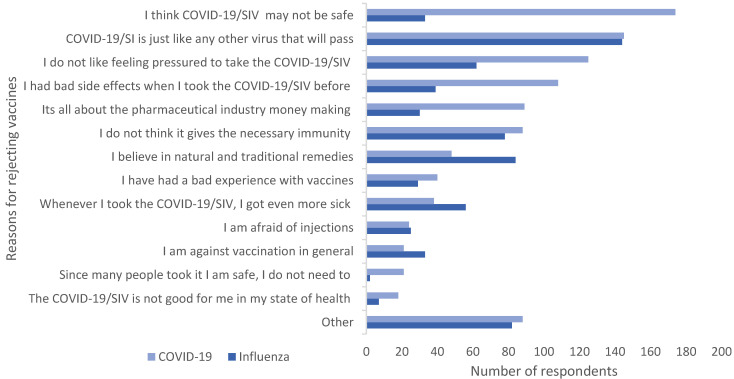
Reasons for rejecting the vaccines. Multiple responses possible. COVID-19/SIV: COVID19/seasonal influenza vaccine.

Figure 1 illustrates respondents’ pre-determined reasons for rejecting the vaccines. The primary reason given by 31.3% (*n* = 174) of respondents for rejecting the COVID-19 vaccine was perceived lack of safety. In addition, 26% (*n* = 144) refused to take the SI vaccine as they did not perceive it as being a threat. Feeling pressured to take the COVID-19-vaccine (22.5%, *n* = 125) was also a popular reason given for rejection.

### 3.3. Participants’ Sources of Information About COVID-19 and SIV Vaccines

**Figure 2:**
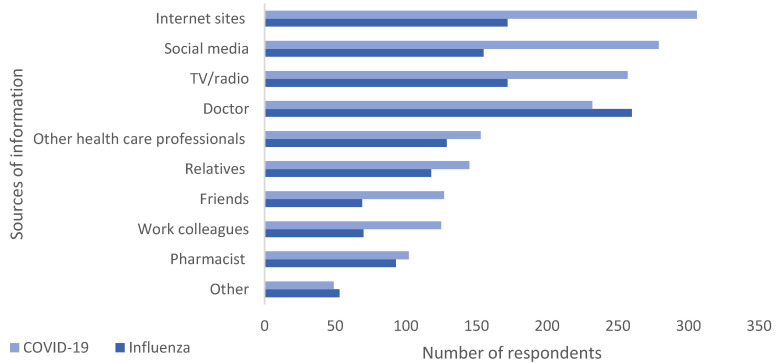
Information seeking behavior. Multiple responses possible.

The sources from which respondents obtained information (Figure 2) about the vaccines differed. Nearly half the respondents (46.8.%, *n* = 260) said that a doctor was the main source for information in the case of the SI vaccine; however, for the COVID-19 vaccine, respondents primarily sought information from internet sites (55.1%, *n* = 306), social media (50.3%, *n* = 279), and TV/radio (46.3%, *n* = 257).

### 3.4. Participants’ Attitudes and Beliefs Toward COVID-19 and SIV Vaccines

Table 2 and Table 3 illustrate pre-determined responses with regards to attitudes and beliefs toward COVID-19 and SIV vaccines, respectively. On further analysis, females were found to be less likely than males to believe that the COVID-19 vaccine helps protect the health of the individual (t = 2.55, df = 191.33, *p* = 0.011). More males were likely to believe that people should be encouraged to take the COVID-19 (t = 3.45, df = 200.43, *p* = 0.001) or the SI vaccines (t = 2.26, df = 198.6, *p* = 0.025). Pearson’s correlation coefficient, r, indicated that the older the age of the respondents was, the less likely they were to be influenced by family and friends regarding their decision to take the SIV (r = −0.104, *p* = 0.015).

One-way ANOVA tests showed significant differences between levels of education and attitudes and behavior toward vaccines. To determine for which education categories the difference was significant, post hoc tests were conducted with *p* values corrected for multiple comparisons using Bonferroni correction. Respondents having tertiary or further education were more likely to believe that the COVID-19 vaccine protected their health (F = 3.03, df = 3548 *p* = 0.01) and gave more importance to advice of health care professionals regarding the COVID-19 vaccine (F = 3.73, df = 3551 *p* = 0.02) than those whose highest level of education was secondary. The opinion of family and friends with regards to the SI vaccine (F = 3.86, df = 3549, *p* = 0.023) and the COVID-19 vaccine (F = 3.20, df3549 *p* = 0.02) mattered less to respondents with a tertiary education than those whose highest level of education was post-secondary.

Independent sample t-tests revealed that respondents who were health care professionals were more likely than other respondents to believe that the SI vaccines will protect the health of individuals who take it (F = 9.02, df = 549, *p* < 0.001), that people should be encouraged to take the SI vaccine (F = 3.79, df = 547, *p* = 0.003), and that they were more likely to value the advice of health care professionals regarding the effectiveness of the SI vaccine (F = 3.80, df = 206, *p* = 0.032). Health care workers were less likely to allow the opinion of family and friends to influence their decision of whether to take the COVID-19 vaccine (F = 6.04, df = −212, *p* < 0.001).

Individuals with chronic conditions were more likely to believe that the COVID-19 vaccine (F = 1.11, df = 209, *p* = 0.018) and the SI vaccine (F = 3.36, df = 216, *p* = 0.02) helped protect the health of the individuals who take it. Additionally, they were also more in favor of individuals being encouraged to take the SI vaccine (F = 1.38, df = 210, *p* = 0.02).

Those who took the COVID-19 vaccine or were planning on taking it and those who took the SI vaccine or intended to take it were more likely to believe that taking the COVID-19 vaccine (F = 8.26, df = 208, *p* < 0.001) and SI vaccine (F = 11.5, df = 197, *p* < 0.001) protects the health of the individuals who take them. The latter were also more likely to agree that individuals should be encouraged to take the COVID-19 (F = 3.10, df = 197, *p* < 0.001) and SI (F = 11.38, df = 206, *p* < 0.001) vaccines, and they were more likely to value the advice of health care professionals regarding COVID-19 vaccine (F = 13.83, df = 209, *p* < 0.001) and SI vaccine (F = 10.6, df = 210, *p* < 0.001).

Most participants declared that should another pandemic arise, they would be happy to engage in preventive NPAs. A total of 72.8% (*n* = 404) scored between 8 and 10 on a Likert scale (1—lowest negative score; 10—highest positive score). Health care workers (F = 24.06, df = 263, *p* < 0.001), individuals with chronic conditions (F = 13.9, df = 257, *p* < 0.001), as well as those who took or intended to take either vaccine (F = 12.06, df = 536, *p* < 0.001) were even more in favor of engaging in preventive measures than other participants.

### 3.5. Willingness to Take a Combined COVID-19 and Seasonal Influenza Vaccine

**Figure 3:**
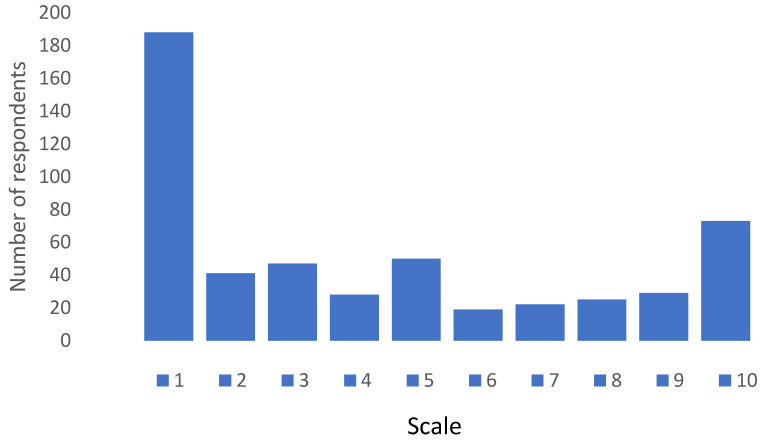
Response pattern regarding willingness to take combined COVID-19 and seasonal influenza vaccine. Scale—1: absolute negative score; 10: absolute positive score.

When respondents were asked if they would be willing to take a combined seasonal influenza and COVID-19 vaccine the response was overwhelmingly negative as can be seen in Figure 3. On a Likert scale ranging from 1—absolute negative score and 10—absolute positive score, 50% of respondents scored 3 or less, whilst only 23% scored between 8 and 10, with a mean score of 4.31 ± 3.28.

A total of 486 (87.6%) respondents provided reasons for the score given.

Thematic analysis of the responses produced three main themes: positive reactions, negative reactions, and mixed or neutral reaction toward the combined vaccine. Table 4 provides an overview of the themes, subthemes and verbatim quotes to support each subtheme.

#### 3.5.1. Positive Reactions: Confidence Rooted in Experience and Logic

Positive responses were more common among individuals who regularly took both vaccines and had positive personal experiences with minimal side effects. There was also a strong trust in science and the medical profession, with several indicating openness to the vaccine contingent on successful clinical trials. This group tended to trust institutions and adopt a rational, pragmatic health behavior model. Participants who reacted positively to a combined vaccine did so primarily due to its convenience, practicality, and perceived efficacy.

Convenience: Participants mentioned that a combined vaccine was more convenient because it meant fewer injections, a single recovery period, and broader accessibility. Those who had taken the vaccines with minimal or no side effects were more likely to be in favor of a combined vaccine (Table 4, q1–q2).

Practicality: Participants recognized the time-saving aspect (‘*hitting 2 birds with 1 stone’*). Practicality was highlighted in terms of efficiency for both patients and public health vaccine programs (Table 4, q3–q6).

Efficacy: Some perceived synergistic effects of combining the vaccines to provide enhanced immunity. They were of the belief that a combination would have a synergistic effect (Table 4, q7).

#### 3.5.2. Negative Reactions: Anchored in Fear, Experience, and Distrust

Negative reactions dominated the discourse, driven by past adverse experiences and health fears. These concerns reflected deep-seated mistrust of the medical system, government policies, and pharmaceutical motivations. There were many negative comments associated with a combined vaccine. Some participants were against one or the other of the vaccines or both. Some others questioned whether it was safe, while others questioned its effectiveness. This profound distrust in institutions and pharmaceutical companies was strongly expressed. The experiences described had high emotional salience. They pointed to perceived betrayal during the pandemic, exacerbated by information overload and mixed messaging. The use of social media and alternative sources may have reinforced these narratives. The emerging subthemes are presented below with the verbatim supporting quotes listed in Table 4.

Health complications: Respondents voiced their concerns about the effect of these vaccines on chronic illness, allergies, and pregnancy complications. They gave anecdotal reports of worsening conditions post-vaccine. Some respondents were concerned that the vaccine could complicate their current conditions (Table 4, q8–q10).

Negative effects on pregnancy and pregnancy outcomes were also a concern (Table 4, q11).

Side Effects: Participants gave detailed accounts of severe reactions, including physical (e.g., shaking, fever) and chronic (e.g., anxiety, menstrual changes), experienced by themselves or people they know after taking the vaccine. Many respondents reported severe adverse reactions from the COVID-19 vaccine, such as fever, extreme shaking, and long-term health issues. These negative experiences made them wary of taking a combined vaccine. Participants who experienced severe health issues post-vaccination, including personal anecdotes of adverse reactions such as increased eczema flare ups, missed menstrual cycles, and other chronic conditions, lead to reluctance in taking further doses because they were afraid that the combined vaccine could give rise to health complications (Table 4, q12–q18).

Effectiveness: Participants said that vaccines did not prevent illness—undermining faith in their purpose. There was concern regarding the effectiveness of the combined vaccine, since both when taking the SI vaccine and COVID-19 vaccine, they contracted the virus that was meant to be prevented (Table 4, q19–q21).

Skepticism and distrust: Strong language was used by participants when they expressed their distrust which suggested that there was manipulation, rushed approvals, and lack of transparency. They had fears about long-term effects (e.g., DNA interference). As expected, skepticism and distrust also featured significantly in the negative comments related to a combined vaccine (Table 4, q22–q27).

There was also reference to the rushed development and approval process of the COVID-19 vaccine and this created general distrust about its safety, long-term effects and ongoing symptoms. The belief that the vaccine was not adequately tested created concerns about the transparency of information provided by the health authorities and pharmaceutical companies (Table 4, q28–q30).

Loss of autonomy: Some participants expressed a desire to preserve individual choice —even if not wholly against vaccines. Some wanted to be free to make the choice of which vaccine to take (Table 4, q31–q32).

#### 3.5.3. Mixed or Neutral Reactions: Caution and Conditional Acceptance

Participants in this group were open to the idea of a combined vaccine but demanded more clarity, data, and professional reassurance. *‘I would need to read some studies regarding the effect of that vaccine beforehand’.* These individuals demonstrated cognitive openness, tempered by risk aversion and uncertainty. They represented a strategic target for public health messaging—likely to respond well to clear, credible, and personalized information.

Some participants were not so categorical about the perceived positive and negative effects of the combined vaccine. They were open to the idea of taking the combined vaccine if there were more studies, more transparency, and possibly the recommendation by a trusted professional. *‘I will consider it but there has to be multiple phases of testing as it will be a new vaccine fighting two viruses at once’.* The following subthemes provided a deeper insight into these respondents’ attitudes.

Need for more information: Participants spoke of the desire for rigorous studies, long-term data, and transparent communication. Individuals who were unsure or had mixed feelings often cited the need for more research and information about the combined vaccine’s safety and efficacy (Table 4, q33–q36).

Recommendations from trusted professionals: Many of the participants in this category were willing to be guided by medical professionals, indicating residual trust in health care, albeit with skepticism. Trusting the source of information was also a deciding factor (Table 4, q37–q39).

## 4. Discussion

The results indicate that in the post-pandemic era, COVID-19 vaccines and SI vaccines are not widely perceived by respondents as having a major role in protecting one’s health. There is still a high degree of skepticism toward the COVID-19 vaccine, while influenza is not considered as a significant threat. Females have an overall more negative attitude toward vaccines than their male counterparts. To our knowledge, these findings offer new insights since research into attitudes and beliefs regarding both the vaccines post-COVID is highly limited.

The general profile and demographic information of respondents provided us with an insight regarding their attitude toward the vaccines and demonstrates that they were not particularly willing to engage with either the SI or COVID-19 vaccines. This is consistent with published data [22,23].

Women are more skeptical toward vaccines as compared to their male counterparts. The present study found that women were less likely to have taken or intended to take either vaccine. They were less likely to be in favor of encouraging the population to take either vaccine. These finding are consistent with other studies looking at vaccine hesitancy and attitudes toward vaccines globally [19,24,25,26]. Toshkov (2023) conducted a study in 27 European countries, including Malta, to understand the reasons behind the gender gap which revealed that women perceived the COVID-19 vaccines as being unsafe, ineffective, and associated with more risks than benefits [27]. Understanding women’s attitudes toward vaccines is important as they can influence the decision to vaccinate their children or even elderly parents. They are also more active on social media than males, so they can influence more people by what they post [28]. Increased educational and behavioral interventions should be designed and targeted specifically at women addressing these issues.

The present study indicates that individuals with chronic conditions have increased coverage of both vaccines and have a more positive attitude toward them, possibly demonstrating that the key public health message with regards to vaccination saving lives and decreasing morbidity in this at-risk group has been effective. The literature is conflicting with regards to COVID-19 and seasonal influenza coverage in people with chronic /long-term conditions. Bulusus (2023) reported that in Washington, DC, USA, the odds of being vaccinated were 1.4 times higher in individuals with no chronic conditions than those with chronic conditions [29]. A pan-European study conducted in 2021 likewise reported that those with chronic conditions were less likely to have declared taking either vaccine [16].

Vaccine uptake by health care workers is important to protect both their health as well as that of the patients whom they treat. A health workforce with low coverage could lead to an increased burden on systems, which already face significant staff shortages [30]. While health care workers demonstrated a relatively positive attitude toward the SI vaccine, no such finding emerged with regards to COVID-19 vaccine, indicating a lower degree of confidence in the COVID-19 vaccine. While vaccine hesitancy amongst health care workers was present during the pandemic, recent studies have shown that this is an evolving situation [31,32,33,34]. Many reasons could account for the fact that health workers have positive attitudes toward SIV. Health care workers may still have concerns about the long-term effects of the COVID-19 vaccines and the risk/benefit ratio in light of evolving virus strains which could lead to the population receiving mixed messages from health care professionals resulting in diminished confidence in public health with a negative impact on vaccine uptake [35,36].

The primary reason for rejecting the COVID-19 vaccines was the belief that it may not be safe. This reasoning is apparent in international studies conducted within a comparable time frame [26,37]. These finding indicate that the concerns for vaccine hesitancy regarding vaccine safety during the pandemic are still present [19]. One of the main factors cited was the fast development of the vaccine, a belief that was well engrained in the population before vaccine roll out began [20]. The European Medicines agency has clearly declared COVID-19 vaccines to be safe and effective [38]. However, a form of cognitive bias may be contributing to this belief, where one may prefer to maintain current or previous belief even though there is evidence indicating a better alternative [39].

The perception that COVID-19 and seasonal influenza are no longer a threat is a dangerous one, since both viruses are still a significant cause of morbidity and mortality, especially in high-risk groups. It is well established that vaccination is the most effective measure to protect against more severe forms of respiratory viral diseases. Respondents may, however, perceive that the risk/benefit scenario has shifted. One of the contributors to this belief, which is stronger for the COVID-19 vaccine, may be linked to participants’ information seeking behavior (Figure 2), where the main information sources for COVID-19 vaccines were the internet and social media making them more exposed to misinformation [40,41]. It is well established that social media is a threat to public health due to numerous factors including, but not limited to, the ability of uninformed individuals to rapidly post information, the effect of bots and social media algorithms [42]. In the case of SI, the doctor, a more reliable source, was the primary source of information.

The uptake of a combined COVID-19 and seasonal influenza vaccine was not generally received positively by the participants of the present study. This is not in line with the findings of other studies both in Europe [43] and the Eastern Mediterranean region [44] where acceptance of a combined vaccine was good with indications that a combined vaccine would increase vaccine uptake. The present study suggests that uptake of the seasonal influenza vaccine would decrease and that there would not be any positive impact on the uptake of COVID-19 vaccine. The qualitative data gathered provide significant insight into the reasons for respondents’ choice response. Acceptance of a combined vaccine is a complex issue, and one that merits further study. Personal experiences play a crucial role in shaping opinions about the combined vaccine. Positive experiences with minimal side effects lead to greater acceptance, while negative experiences and adverse reactions result in skepticism and reluctance. Trust is central to vaccine uptake. The role of trusted professionals is crucial. Where that trust is broken or unestablished, hesitance strives.

Research findings highlight the critical need for policy to be informed by targeted, trust-building, and responsive public health strategies. Public communication campaigns should be tailored to address the specific concerns of distinct population groups, such as women, individuals with chronic illnesses, and vaccine-hesitant health care professionals. Restoring public trust requires transparent communication. Authorities must openly acknowledge potential side effects and address them with honesty, rather than minimizing or dismissing them. Furthermore, health care professionals, particularly doctors and pharmacists, should be equipped with the tools and training to serve as effective, trusted communicators of vaccine-related information. Policies must prioritize the active monitoring of social media and other digital platforms to swiftly counter misinformation and engage with public fears and myths in real time.

The present study has some limitations. The data were gathered through social media and therefore possibly excluded individuals who do not have access to or make use of social media. The prevention of duplicate responses due to the anonymous survey design was not possible. The cross-sectional design of the study limits the determination of causal inferences. As with other web-based surveys, the data collected may be biased in favor of those who are interested in the topic under investigation or who have a personal agenda, for or against, vaccines. Moreover, there is an over representation of female respondents and individuals having a tertiary education; therefore, the sample is not representative of the general population. Since it is not a random sample, the results cannot be generalized to the population and should be interpreted with caution. The fact that the findings seem to be in line with other studies carried out in several other countries is encouraging. Psychometric validation tests for Likert scale items assessing attitudes and beliefs were not conducted since the questions were only loosely based on the Theory of Planned Behavior. Future research on the topic, both qualitative and quantitative, should consolidate the findings emerging from this exploratory study.

These findings need to be interpreted in relation to the belief of a decreased threat to health from these viruses and a possible shift in perceived risk/benefit ratio associated with taking the vaccines. Education and communication campaigns need to be designed and targeted to address the concerns of specific groups providing balanced and transparent information. With the known threat of a future pandemic, it is of utmost importance that public health institutions learn from past experience and are also sensitive to trends in societal attitudes and behaviors to enact effective health policies.

## Figures and Tables

**Table 1:** Participants’ demographic profile and vaccine uptake for season 2023–2024.

			Took or Intended to Take COVID-19 Vaccine in Season 23–24		Took or Intended to Take Seasonal Influenza Vaccine in Season 23–24	
	N	%	N	%	N	%
Gender						
Male	126	22.7	35	6.3	52	9.4
Female	427	76.9	81	14.6	128	23.0
Prefer not to say	1	0.2	1	0.2	1	0.2
Age (years)						
16–19	49	8.8	10	1.8	18	3.2
20–29	87	15.7	11	2	21	3.8
30–39	90	16.2	16	2.9	21	3.8
40–49	118	21.3	18	3.2	27	4.9
50–59	127	22.9	30	5.4	45	8.1
60–69	51	9.2	18	3.2	24	4.3
70–79	26	4.7	8	1.4	18	3.2
80 and over	7	1.3	6	1.1	6	1.1
Marital status						
Single	117	21.1	26	4.7	38	6.8
In a relationship/married	422	76	74	13.3	116	21
other	16	2.9	15	2.7	23	4.1
Level of education						
Primary	2	0.4	2	0.4	2	0.4
Secondary	76	13.7	15	2.7	27	4.7
Post-secondary	110	19.8	21	3.8	41	7.4
Tertiary/further education	367	66.1	79	14.2	110	19.8
Health care worker (HCW)						
Yes	121	21.8	22	4	45	8.1
No	432	77.7	95	17.1	134	24.14
Chronic/long term condition						
Yes	126	22.7	40	7.2	55	10
No	427	77.0	77	13.9	125	22.5
Taken COVID-19 vaccine this year						
Yes	90	18.2	-	-	-	-
No	447	80.5	-	-	-	-
If ‘No’, do you intend to take it						
Yes	27	4.8	-	-	-	-
No	421	75.8	-	-	-	-
Taken influenza vaccine this year						
Yes	144	25.9	-	-	-	-
No	399	71.9	-	-	-	-
If ‘No’, do you intend to take it						
Yes	36	6.4	-	-	-	-
No	357	64.3	-	-	-	-

Percentages do not add up to 100% due to missing data.

**Table 2:** Attitudes and beliefs toward the COVID-19 vaccine.

	Mean (SD)	Minimum	Q1	Median	Q3	Maximum
I believe that the COVID-19 vaccine helps protect the health of the people who take it	6.55 (2.90)	1	5	7	9	10
I believe that people should be encouraged to take the COVID-19 vaccine	5.72 (3.13)	1	3	6	8	10
The opinion of my family and friends is important in my decision to take the COVID-19 vaccine	3.98 (2.88)	1	1	3	6	10
I value the advice of health professionals regarding the effectiveness of the COVID-19 vaccine	7.09 (2.84)	1	5	8	8	10
It is easy for me to get the COVID-19 vaccine	7.5 (2.7)	1	6	8	10	10

Q1: 1st Quartile; Q3: 3rd Quartile.

**Table 3:** Attitudes and beliefs toward the seasonal influenza vaccine.

	Mean (SD)	Minimum	Q1	Median	Q3	Maximum
I believe that the seasonal influenza vaccine helps protect the health of the people who take it	6.76 (2.81)	1	5	8	9	10
I believe that people should be encouraged to take the seasonal influenza vaccine	6.36 (2.99)	1	4	7	9	10
The opinion of my family and friends is important in my decision to take the seasonal influenza vaccine	3.71 (2.83)	1	1	3	6	10
I value the advice of health professionals regarding the effectiveness of the seasonal influenza vaccine	7.08 (2.82)	1	5	8	10	10
It is easy for me to get the seasonal influenza vaccine	7.77 (2.68)	1	6	9	10	10

Q1: 1st Quartile; Q3: 3rd Quartile.

**Table 4:** Themes and subthemes emerging from thematic analysis of respondents’ comments on a combined COVID-19 and seasonal influenza vaccine.

Theme	Subtheme	Quotes
Positive reactions rooted in experience and logic	Convenience	‘*More convenient, less needles and only one sore spot on one arm’ (q1)* *‘Available in health centres and stocked by pharmacies’ (q2)*
	Practicality	*‘I would recover from any side effects in one go’ (q3)* *‘Saves time and money’ (q4)* *‘Less time consuming. Only 1 recovery period instead of double’ (q5)* *‘Makes sense logistically and public health-wise’ (q6)*
	Efficacy	*‘Combined vaccines offer better immunity’ (q7)*
Negative reactions anchored in fear, experience and distrust	Health complications	*‘I am somewhat prone to allergies, so I would rather take the vaccines one at a time’ (q8)* *‘I have to be fit/ healthy to take any vaccine, therefore, since one doesn’t know for sure, I won’t take it’ (q9)* *‘Some studies seem to indicate that people who suffer with heart problems have seen their heart problem regress at a faster rate. So, I would never take a combo vaccine.’ (q10)* Negative effects on pregnancy and pregnancy outcomes were also a concern. *‘One of my friends, was pregnant and her employer told her that her must take vaccine, her baby had problems, even her. I didn’t get any of vaccines and I had 2 days temperature of 38. And also I was pregnant then, but I refused be vaccinated.’ (q11)*
	Side effects	*‘I am not comfortable taking the COVID vaccine due to the negative effect the last booster dose had on me (sudden rapid fever and extreme shaking) I have however been taking the influenza vaccine for over 10 years and believe that it is more well researched.’ (q12)* *‘I got Bigeminy and low blood count after the COVID vaccine, I will not take anymore. The flu vaccine works well with me, I take this annually.’ (q13)* *‘From when I took the COVID vaccines, the 3 of them, I got extreme anxiety and weak legs at 28 years old sometimes I feel my heartbeat is going fast and I feel dizzy in an instance and weak and need sleep’ (q14)* *‘COVID jab not interested I believe my chronic illness now is due to jabs I took’ (q15)* *‘If I experience an adverse event I would like to know exactly which one caused it’ (q16)* *‘Too many side effects and possible long-term complications’ (q17)* *‘There have been significant adverse effects with both vaccines particularly the influenza vaccine hence a combination of the two might also induce severe adverse effects and furthermore their efficacy is short-lived and would likely require a booster shot a few months after’ (q18)*
	Effectiveness	*‘Was bad when I took vaccine on 3rd time and in any case it was then I caught the COVID just the same’ (q19)* *‘Each time I took the flu vaccine I was always terribly sick with the flu’ (q20)* *‘The influenza vaccine—I have taken this occasionally in the past and have not found it to be effective and therefore have avoided taking it’ (q21)*
	Skepticism/distrust	*‘mRNA vaccines are one big human experiment. We do not yet know the long-term effects. Much has been hidden. Some have died with the vaccine. Others have health problems. The literature shows that they had more side effects than benefits. It’s a money-making business and we can’t rely on the manufacturers to reveal all the effects. Re the influenza vaccine, many who take it report feeling ill after’ (q22)* ‘*I want to stop being a Guinea pig to pharmaceutical companies that are amplifying fear to help them make money’ (q23)* *‘COVID vaccine I think was invented in a very short period of time and we do not know the consequences that it might have on our health’ (q24)* ‘*The COVID-19 vaccine was never safe and effective to begin with due to a lack of long-term studies on it. As a result, we are seeing an excessive death rate and terminal illnesses caused by the said vaccine’ (q25)* *‘After the pandemic I lost trust in the health system as it was clear that all they wanted was to make money’ (q26)* *‘Too much information I’ve felt overwhelmed, confused and finally sceptical on the matter of vaccine. I would not opt to get vaccinated anymore’ (q27)* *‘Rushed approval at EU level very sceptical about it’s benefits and am afraid in long term will cause more harm than good in healthy individuals who took it. I categorically refuse to take any further doses of it.’ (q28)* *‘I’m a little concerned about claims of such vaccines on any ‘reprogramming’ effect or interference with my DNA constitution’ (q29)* *‘The realities coming out in scholarly papers regarding COVID-19 are deeply distressing’ (q30)*
	Loss of Autonomy	*‘I want the flexibility to choose which one or both I need to take.’ (q31)* *‘I want to always be able to decide whether I want to take the COVID vaccine or not’ (q32)*
Mixed or neutral reactions indicating caution and conditional acceptance	Need for more information	*‘Before it is tested properly, medically approved and licensed with the right amount of research, I would have my doubts about taking a combined vaccine’ (q33)* *‘I would need more information on the vaccine’ (q34)* *‘I have to educate myself about it. Not very keen on taking COVID 19 vaccine again’ (q35)* *‘Would prefer to have more information about such a combination’ (q36)*
	Recommendations from trusted professionals	*‘If the vaccine will be safe with not much side effects, I would take it. It should be approved with doctors’ (q37)* *‘I will take it if I am convinced by medics that it is safer to do so for my vulnerable family’ (q38)* *‘I did not see much in the local media that assured me that the COVID vaccine is safer and (that) my “side effects” were not caused by the vaccine. One would believe that now that more time has passed, there is much more information available on the safety of the vaccine compared to before. Therefore, I would like to see more information backed by serious scientific studies carried out by reputable organizations that have been verified.’ (q39)*

## Data Availability

The original contributions presented in this study are included in the article. Further inquiries can be directed to the corresponding author(s).
